# Low Frequency of Viral Respiratory Tract Infections During Family-Centered Neonatal Intensive Care: Results of a Prospective Surveillance Study

**DOI:** 10.3389/fped.2020.606262

**Published:** 2020-11-16

**Authors:** André Kidszun, Anna Neurohr, Britta Gröndahl, Susanne Tippmann, Daniel Schreiner, Julia Winter, Seyed Hamidreza Mahmoudpour, Stephan Gehring, Eva Mildenberger

**Affiliations:** ^1^Department of Neonatology, University Medical Center, Mainz, Germany; ^2^Department of Pediatric Infectious Diseases, University Medical Center, Mainz, Germany; ^3^Institute of Medical Biostatistics, Epidemiology, and Informatics, University Medical Center of the Johannes Gutenberg University Mainz, Mainz, Germany; ^4^Center for Thrombosis and Hemostasis, University Medical Center of the Johannes Gutenberg University Mainz, Mainz, Germany

**Keywords:** family-centered, neonatology, infection, virus, surveillance

## Abstract

**Background:** Viral respiratory tract infections (VRTI) may cause severe respiratory and sepsis-like symptoms in infants hospitalized in the neonatal intensive care unit (NICU). Little is known about the frequencies of VRTI in relation to visiting policies in the NICU.

**Objective:** Aim of this study was to evaluate the frequency of symptomatic and asymptomatic VRTI in our family-centered NICU.

**Methods:** This was a 12-month, prospective, observational study from February 2018 to January 2019. Infants hospitalized ≥72 h were eligible for the study. To determine the frequency of VRTI, multiplexed point-of-care testing (mPOCT) of symptomatic infants was combined with a weekly screening of all infants. Our 10-bed NICU is 24/7 open to families and visitors. The number of simultaneous visitors is restricted to two per patient. Parents and visitors are instructed in hand hygiene and advised to avoid visits in cases of respiratory illness. Siblings irrespective of age may visit the NICU following a physical check-up.

**Results** Multiplexed point-of-care testing (71 symptomatic episodes) combined with the weekly screening (272 episodes) yielded in 21 positive samples from 2 of the 67 infants enrolled in the study. Both infants were first detected during symptomatic episodes. Rhino-/enterovirus were detected in all cases.

**Conclusion:** Respiratory viruses were detected during symptomatic and asymptomatic episodes but affected <3% of infants enrolled in the study. In our unit, a low frequency of VRTI was attained despite adherence to family integrated care including liberal visiting policies for younger siblings.

## Introduction

Viral respiratory tract infections (VRTI) have been described to cause severe respiratory and sepsis-like symptoms in infants hospitalized in the neonatal intensive care unit (NICU) (1–3). During birth hospitalization, VRTI have also been associated with prolonged hospital stay, increase in home oxygen use and elevated rates of bronchopulmonary dysplasia (4, 5). However, and despite a growing body of literature, the magnitude of the problem is not sufficiently known (6). Evidence concerning frequency and impact of VRTI in the NICU is sparse and conflicting. While in one study, the incidence of VRTI was more than 50% in neonates <33 weeks of gestation (5), others only found four virus-positive cases in a cohort of 93 preterm infants (7). Study methodology varied markedly between available publications and so far only one study used a comprehensive, albeit blinded surveillance design (5). In particular, the significance of clinically asymptomatic VRTI remains unclear. Overall, evidence appears insufficient to inform clinicians and policy makers concerning indications for virus testing and visiting policies (6, 8). Aim of this study was to evaluate the frequency of symptomatic and asymptomatic VRTI in our family-centered NICU.

## Methods

This was a 12-month, prospective, observational study from February 2018 to January 2019. Infants hospitalized postnatally ≥72 h in the NICU were eligible for the study.

Surveillance of VRTI consisted of multiplexed point-of-care testing (mPOCT) of symptomatic infants, which was combined with a weekly screening of all infants. Infants were considered symptomatic if they were started on intravenous antibiotics due to suspicion of a bacterial infection or if they exhibited new and not otherwise explicable respiratory symptoms (i.e., nasal congestion, FiO_2_ increase of ≥0.1, increased pressure support or intubation).

Virus samples were collected via flocked nasopharyngeal swabs with a sterile viral transport medium and analyzed with the BioFire® FilmArray® Respiratory RP2 Panel (mPOCT) and an in-house multiplexed PCR (weekly screening). The RP2 Panel detects adenovirus, coronaviruses (HKU1, NL63, 229E, and OC43), human metapneumovirus, human rhinovirus/enterovirus, influenza (A, A/H1, A/H3, A/H1-2009, and B), parainfluenza virus 1–4 and respiratory syncytial virus. Clinicians performed mPOCT as soon as possible after sample collection and were not blinded to the test results. Analysis was performed in the NICU. Results were usually available within 2–6 h. At each individual episode, clinicians were asked to indicate whether they felt that the test results were useful or not regarding hygiene measures, antibiotic therapy, or establishing a diagnosis. Clinicians were blinded to the results of the weekly screening tests. The 19-valent in-house multiplexed PCR method detects a similar range of viruses and was performed as previously described (9). Infants' clinical characteristics were recorded as was the frequency of parents' and siblings' visits.

Our 10-bed NICU is a tertiary care facility that is located near to the delivery suite and the maternity ward. The unit primarily cares for preterm infants with a birthweight <1,500 g and more mature infants with respiratory or circulatory support. The majority of neonates admitted to the NICU are transferred to a 20-bed special care unit following initial stabilization. Most infants are inborn and are admitted immediately after birth. However, the unit also accepts infants transferred from other hospitals, readmissions from the special care unit and in some cases, infants after discharge home. The NICU consists of three patient rooms, a 5-bed, a 4-bed, and a single room. The shared rooms are rather small with an average distance between the incubators of 2–3 m. For this reason, no permanent rooming-in for parents or families is possible. Nevertheless, we encourage mothers and fathers to do kangaroo care whenever, and as long as possible, also during night time. Parents have a lounge area in close proximity to the NICU. A separate family home is provided for overnight stays. The unit makes comprehensive efforts to integrate parents and to support their role as primary caregivers for their infants. Areas of family-centered care include, but are not limited to, communication, involvement in care, peer-to-peer support, and parent education.

The NICU is 24/7 open to families and visitors (e.g., other family members, close friends). The number of simultaneous visitors is restricted to two per patient. Parents and visitors are instructed in hand hygiene and advised to avoid visits in cases of respiratory illness. Siblings irrespective of age may visit the NICU following a physical check-up by the NICU physicians at least once a week. The duration of sibling visits is not restricted. Siblings are allowed to stay with their parents during kangaroo care, but we usually allow only parents to do kangaroo care.

Prompt isolation measures are implemented in virus-positive cases, which include single room accommodation if possible, cohorting of positive infants, care with gloves and gowns, and a detailed parent's instruction in hygiene measures.

The local ethics committee of the Rhineland-Palatinate Medical Association approved this study. Legal guardians of the infants provided written informed consent before inclusion in the study.

## Results

During the study period, 366 infants were admitted to the NICU. Of those, 70 were eligible for the study. Three parents declined participation, so 67/70 infants (96%) were finally enrolled and analyzed. Table 1 summarizes the infants' characteristics.

**Table 1:** Demographic characteristics.

**Characteristic**	
No. of enrolled infants	67
Gestational age, week, median (IQR)	29.4 (26.4–32.9)
Gestational age, ≤ 32 weeks, *n* (%)	47 (70.1)
Gestational age, ≤ 28 weeks, *n* (%)	24 (35.8)
Female, *n* (%)	33 (49.3)
Birth weight, mean (SD), g	1,524.8 ± 980.6
nCPAP, *n* (%)	58 (86.6)
Intubation, *n* (%)	47 (70.1)
Bronchopulmonary dysplasia, *n* (%)	17 (25.4)
Discharge on home oxygen, *n* (%)	2 (3)
Death before discharge, *n* (%)	1 (1.5)

*nCPAP, nasal continuous airway pressure; IQR, interquartile range; SD, standard deviation*.

In total, 75 symptomatic episodes and 272 weekly screening episodes were recorded. Complete data were available for 71 of the 75 (94.7%) symptomatic and all 272 (100%) screening episodes. Via mPOCT, two infants were diagnosed with VRTI. Infant A was born at 26+1 weeks of gestation with a birth weight of 1100g. He was tested positive in May 2018 on day of life (DOL) 63. Major clinical symptoms were increased work of breathing and re-introduction of nasal continuous airway pressure. Infant B was born at 25+6 weeks of gestation with a birth weight of 410 g. He was first tested positive in October 2018 on DOL 25. His clinical symptoms were rising FiO_2_ on nasal continuous airway pressure and frequent apneas. Subsequently, that infant was repeatedly tested positive during several symptomatic episodes (9 positive samples) and also during the weekly screening (11 positive samples). Human rhinovirus/enteroviruses were detected in all cases.

No new VRTI were diagnosed during asymptomatic episodes.

Frequency of parents' visits was recorded in 233 of the 272 weekly screening episodes. Daily parents' visits were seen during 208 of those 233 (89.3%) episodes. Visits of siblings younger than 12 years of age were recorded in 25 of 233 (10.7%) episodes, respectively. During 21 weeks of the 52-week study period, at least one sibling younger than 12 years of age was present in the NICU.

Clinicians' perceptions of usefulness of mPOCT results are presented in Table 2. All positive virus tests were considered useful concerning isolation measures, whereas overall usefulness of mPOCT, especially when negative, was judged low.

**Table 2:** Clinician's evaluation of mPOCT results.

**Condition**	mPOCT results judged as useful [all results^*^]	**Negative mPOCT results judged as useful**	**Positive mPOCT results judged as useful**
Hygiene measures	21/68 (30.9%)	13/60 (21.7%)	8/8 (100%)
Antibiotic treatment	10/68 (14.7%)	7/60 (11.7%)	3/8 (37.5%)
Diagnostic certainty	19/68 (27.9%)	14/60 (23.3%)	5/8 (62.5%)

* *Data available for 68 of 71 episodes; multiple answers possible*.

*mPOCT, multiplexed point-of-care test*.

## Discussion

In this study, VRTI were rare in the NICU. This is in contrast to the findings of Bennett et al. (5) who detected VRTI in many of their NICU patients, but similar to those of Caserta et al. (7) and Shui et al. (10).

Our findings suggest that routine surveillance is not warranted in asymptomatic infants whereas symptomatic infants should be tested for possible VRTI. That may include infants with new respiratory symptoms, respiratory deterioration, and infants with suspected bacterial sepsis (2, 11, 12).

Our study also suggests that surveillance of VRTI might play a role in the prevention of viral spread. In the study of Bennett et al., clinicians were blinded to the results of the virus tests and so unable to isolate or cohort positive infants. However, because viruses may persist for weeks, these measures may be crucial for the prevention of virus transmissions or outbreaks in the NICU.

Other factors that might explain differing frequencies of virus-positive infants may be the size of the NICU and local visiting policies. Previous evidence from retrospective analysis suggested that restriction of young sibling visitation is associated with less respiratory syncytial virus positive infants in the NICU (13). In Bennet's study, children younger than <17 years were even excluded from visiting the NICU (5). In contrast, in our family-centered setting, the frequency of VRTI was low and virus transmission did not occur despite one infant being virus-positive for several weeks. In order to maintain family-centered care and foster family–child interaction, we suggest that virus testing, isolation of positive cases and enhancing hygiene measures should be preferred over restricting visiting policies. Our results are in line with those of Horikoshi et al. (14) whose retrospective analysis detected no correlation between sibling visits and the rate of nosocomial viral infections in a Japanese NICU. In our study, clinicians' judgements supported this assumption. Although clinicians judged mPOCT as unhelpful with respect to antibiotic treatment and establishing a diagnosis, all positive test results were considered helpful regarding hygiene measures.

The study's main limitations are its single-center design and limited sample size. Larger units will have more visitor traffic and more health care personnel involved. In addition, the study period was outside an epidemic viral season, with an average viral community burden. Previous observational data indicated that restricting visitors to NICUs may reduce the incidence of VRTI during pandemic times (15). Hence, more large-scale studies are needed across periods of high community burden to establish if our findings are generalizable.

In summary, VRTI affected <3% of NICU infants enrolled in this study. In our unit, a low frequency of VRTI was attained despite adherence to family-centered care including liberal visiting policies for younger siblings. Testing for VRTI may help preventing viral spread from positive infants as it allows early detection and prompt implementation of isolation and enhanced hygiene measures.

## Data Availability Statement

The raw data supporting the conclusions of this article will be made available by the authors, without undue reservation.

## Ethics Statement

This study involving human participants was reviewed and approved by Ethics committee of the Rhineland-Palatinate Medical Association. Written informed consent to participate in this study was provided by the participants' legal guardian/next of kin.

## Author Contributions

AK: methodology (lead), conceptualization (equal), writing—original draft (lead), writing—review, and editing (equal). AN: resources (supporting), formal analysis (supporting), writing—original draft (supporting), writing—review, and editing (equal). BG: resources (lead), software (lead), writing—review, and editing (equal). ST: resources (supporting), formal analysis (supporting), writing—review, and editing (equal). DS: conceptualization (supporting), writing—review, and editing (equal). JW: validation (supporting), writing—review, and editing (equal). SM: formal analysis (lead), writing—review, and editing (equal). SG: resources (supporting), conceptualization (supporting), methodology (supporting), writing—review, and editing (equal). EM: supervision (lead), validation (lead), methodology (equal), conceptualization (equal), writing—original draft (supporting), writing—review, and editing (equal). All authors contributed to the article and approved the submitted version.

## Conflict of Interest

The authors declare that the research was conducted in the absence of any commercial or financial relationships that could be construed as a potential conflict of interest.
